# When fast logic meets slow belief: Evidence for a parallel-processing model of belief bias

**DOI:** 10.3758/s13421-016-0680-1

**Published:** 2016-12-27

**Authors:** Dries Trippas, Valerie A. Thompson, Simon J. Handley

**Affiliations:** 1 Center for Adaptive Rationality, Max Planck Institute for Human Development, Berlin, Germany; 2 Department of Psychology, University of Saskatchewan, Saskatoon, Saskatchewan Canada; 3 Faculty of Human Sciences, Macquarie University, Sydney, NSW Australia

**Keywords:** Deductive reasoning, Conflict detection, Dual process theory, Logic, Belief

## Abstract

Two experiments pitted the default-interventionist account of belief bias against a parallel-processing model. According to the former, belief bias occurs because a fast, belief-based evaluation of the conclusion pre-empts a working-memory demanding logical analysis. In contrast, according to the latter both belief-based and logic-based responding occur in parallel. Participants were given deductive reasoning problems of variable complexity and instructed to decide whether the conclusion was valid on half the trials or to decide whether the conclusion was believable on the other half. When belief and logic conflict, the default-interventionist view predicts that it should take less time to respond on the basis of belief than logic, and that the believability of a conclusion should interfere with judgments of validity, but not the reverse. The parallel-processing view predicts that beliefs should interfere with logic judgments only if the processing required to evaluate the logical structure exceeds that required to evaluate the knowledge necessary to make a belief-based judgment, and vice versa otherwise. Consistent with this latter view, for the simplest reasoning problems (modus ponens), judgments of belief resulted in lower accuracy than judgments of validity, and believability interfered more with judgments of validity than the converse. For problems of moderate complexity (modus tollens and single-model syllogisms), the interference was symmetrical, in that validity interfered with belief judgments to the same degree that believability interfered with validity judgments. For the most complex (three-term multiple-model syllogisms), conclusion believability interfered more with judgments of validity than vice versa, in spite of the significant interference from conclusion validity on judgments of belief.

**Electronic supplementary material:**

The online version of this article (doi:10.3758/s13421-016-0680-1) contains supplementary material, which is available to authorized users.

The effects of beliefs on logical reasoning are pervasive and have been investigated for almost nine decades (Wilkins, 1929). The believability of conclusions influences how arguments are evaluated across a wide range of paradigms. Believable conclusions are deemed more acceptable than unbelievable ones regardless of logical validity (Evans, Barston, & Pollard, 1983), regardless of the strength of the arguments (Stanovich & West, 1997), and regardless of whether the task involves formal or informal reasoning (Thompson & Evans, 2012). The goal of the current paper is to test predictions from two competing theoretical accounts of belief bias in deductive reasoning, both of which can be placed under the wider meta-theoretical framework of dual process theories of reasoning.

According to dual process theory, two types of qualitatively different cognitive processes can be distinguished: Type 1 processes are autonomous and Type 2 processes require working memory (Evans & Stanovich, 2013a). According to the default-interventionist instantiation of dual process theory, Type 1 processes cue default responses which may then be overridden by Type 2 processes. The default-interventionist account further assumes that beliefs are accessible to Type 1 processing, but that accurate logical reasoning requires Type 2 processing (Evans & Stanovich, 2013b). Consequently, belief effects arise because the (generally faster) Type 1 processes substitute an answer based on belief for one based on logical validity (Evans & Curtis-Holmes, 2005).

Recently, however, theorists have acknowledged that this characterization is too simple for a number of reasons (see e.g., Kruglanski & Gigerenzer, 2011). Equating Type 1 processes with bias and Type 2 processing with normative reasoning is a fallacy (Elqayam & Evans, 2011; Evans & Stanovich, 2013a; Thompson, Prowse Turner, & Pennycook, 2011): Type 1 processes produce errors on some occasions and correct responses on others, as do Type 2 processes. This is empirically supported by evidence that judgments based on formal norms such as logic and probability (traditionally equated with Type 2 processing) may be made quickly and implicitly, suggesting that these judgments may, in some instances, arise from Type 1 processes. For example, recent evidence suggests that some types of simple logical arguments are processed autonomously (Bago & De Neys, 2017; Morsanyi & Handley, 2012; Trippas, Handley, Verde, & Morsanyi, 2016; though see Klauer & Singmann, 2013). Similarly, although belief judgments have been shown to be made rapidly and accurately, it is well established that these involve some form of inferential processing distinct from pure fact-retrieval from memory (Reder, 1982). Furthermore, the automaticity of belief judgments depends on various mediating factors such as mind-set and task demands (Wiswede, Koranyi, Mueller, Langner, & Rothermund, 2013). A review of the available evidence suggests that occasionally belief judgments may require considerable time and effort (cf., Handley & Trippas, 2015), suggesting that here too a pure classification in terms of Type 1 or Type 2 processing is overly simplistic. Taken together, these data support models in which logical and belief-based processing is initiated simultaneously (De Neys, 2012, 2014; Handley & Trippas, 2015; Pennycook, Fugelsang, & Koehler, 2015; Sloman, 2014).

Direct evidence for this position comes from a pair of studies by Handley, Newstead, and Trippas (2011) and Pennycook, Trippas, Handley, and Thompson (2014). Handley and colleagues instructed reasoners to evaluate conclusions to very simple logical arguments of the modus ponens form (i.e., if p, then q; p, therefore q). The novel element of the task was that they were asked to provide one of two judgments: on one half of the trials, participants had to evaluate the validity of the conclusion (as is traditionally done), but on the other half, they had to evaluate the believability of the conclusion (as has been investigated extensively in research on truth verification, cf. Reder, 1982). When the two sources of information conflicted, it was found that the validity of the syllogism interfered with the ability to make belief judgments, as evidenced by higher error rates and longer response times for conflict than non-conflict problems. This pattern would not be expected if judgments of validity took longer or were more difficult than judgments of belief. Pennycook and colleagues replicated this finding using a completely different task, modelled on Tversky and Kahneman’s (1973) base-rate task. Participants were provided with the base-rate probability of category membership (e.g., 5% of the people in this sample are engineers and 95% are lawyers) and a personality description of an individual (e.g., John is a great computer programmer and loves board games). Again, when the two sources of information conflicted, the base-rate information interfered with making belief-based judgments, which is inconsistent with the view that the latter form a fast, default response (Kahneman 2011).

Collectively, these data seem to support parallel processing (De Neys, 2012; Handley & Trippas, 2015; Pennycook et al., 2015; Sloman, 2014) in that multiple relevant problem features (e.g., structure and belief content) may be processed simultaneously. In the case where both problem aspects can be assessed in a relatively simple way, they cause mutual interference. However, in cases where one or the other response requires more complex processing, an asymmetry should arise (Handley & Trippas, 2015). According to the parallel-processing model, it is the complexity of the relevant problem features that determines response accuracy and speed. Logical judgments superseded belief judgments in the cases presented by Handley et al. (2011) due to the relative simplicity of the logical structure versus the somewhat more moderate complexity of the belief judgments. This directly implies that as the logical judgments become more complex, the interference should reverse – with conflict affecting logic judgments more than belief judgments.

In support of this hypothesis, some studies show that the extent of belief bias observed varies as a function of the logical complexity of the problem – typically characterized as the number of mental-models that need to be evaluated to determine the validity of an inference (see e.g., Johnson-Laird, 2001). For example, logical problems that can be solved by constructing only a single representation of the premises show less belief-bias than more complex problems – that is, arguments which according to mental-model theory require the construction of up to three representations to definitively determine their logical validity^1^ (Klauer, Musch, & Naumer, 2000; Newstead, Pollard, Evans, & Allen, 1992; Oakhill, Johnson-Laird, & Garnham, 1989; Trippas, Handley, & Verde, 2013). The fact that belief bias tends to be reduced on simpler problems is consistent with the hypothesis that judgments of validity are completed more quickly and thus that beliefs have less of an opportunity to interfere with a rapidly generated logical response. On the other hand, the evidence that belief bias increases with complexity is not always consistent (Evans & Pollard, 1990).

The goal of the current paper is to provide a direct test of the complexity hypothesis by varying the difficulty of the logical task, and asking participants to evaluate logical validity and conclusion believability. We predicted that the degree to which logical validity and belief judgments interfere with each other will depend on the complexity of the processes required to render them. If the logical structures are extremely simple, then we expected to replicate past findings and show that validity interferes more with belief-judgments than vice versa (Handley et al., 2011). As logical complexity increases, this asymmetry should be reduced, and in fact, should be reversed for the most complex logical problems, where believability should interfere more with logic judgments than vice-versa.

## Experiment 1

In Experiment 1, our aim was to replicate and extend the findings by Handley et al. (2011) to a set of more difficult conditionals (modus tollens: If p, then q; not q, therefore not p). Participants were given a set of problems that included both modus ponens and modus tollens inferences and asked to judge whether the conclusion was logically valid half the time or believable the other (see also Johnson-Laird & Byrne, 2002, for a mental-models account of how people reason about such conditional inferences). On half of the trials, logical analysis and belief analysis produced the same response (no-conflict trials: i.e., valid-believable and invalid-unbelievable) and on the other half, they produced different responses (conflict trials: i.e., valid-unbelievable and invalid-believable). The modus ponens trials were expected to replicate Handley et al. (2011) in that logic-belief conflicts should have a greater impact on belief judgments than logic judgments. Whereas performance with modus ponens is usually quite high, accuracy is lower for modus tollens (Wason, 1968; see Evans, Newstead, & Byrne, 1993 for review). Thus, for modus tollens, we predict the asymmetry in complexity to either be reduced or to reverse direction.

### Method

#### Participants

Forty-five undergraduate psychology students from Plymouth University (UK) or the University of Saskatchewan (Canada) participated in exchange for course credit. Thirty-two participants were female and 13 were male (age range = 18–35 years, *M* = 19).

#### Design, materials and measures

We used a 2 (belief-logic conflict: conflict vs. no conflict) × 2 (instructions: logic vs. belief) × 2 (argument type: modus ponens vs. modus tollens) within subjects design. We created four lists containing 64 arguments each, half of which were modus ponens and half of which were modus tollens, based on 32 distinct item content themes (see Table 1 for examples). We crossed logical validity and conclusion believability to create 16 conflict (valid-unbelievable, invalid-believable) and 16 no-conflict (valid-believable, invalid-unbelievable) items within each argument type. Half of the problems were presented under logic instructions and half were presented under belief instructions. Item contents were counterbalanced by using only half of the themes per item list, half of which were used to create modus ponens problems, and half to create modus tollens problems. Within each argument type each theme was presented four times, once in each conflict by instruction cell. Problem contents were taken and extended from Handley et al. (2011, Exp. 5). We measured accuracy, response time, and confidence on each trial. On each trial, the major (conditional) premise was presented. Upon pressing the spacebar the major premise disappeared and the minor (categorical) premise appeared, as did the conclusion and the response options. The response options acted as the instructional cue: under logic instructions, the response options were “valid” and “invalid”; under belief instructions, the response options were “believable” and “unbelievable.” Responses were made by pressing the s-key (valid/believable, depending on instructions) or the k-key (invalid/unbelievable). After each response, we asked the participants to indicate how confident they were that their response was correct on a scale from 1 (guess) to 3 (certain). We also analyzed confidence ratings^2^ as they have been shown to reflect conflict, thus potentially providing converging evidence for the accuracy and response time data (Johnson, Tubau, & De Neys, 2016). There were 16 practice trials with feedback (not analyzed), and 64 experimental trials (presented in a randomized order for each participant).

**Table 1:** Experiment 1: Examples of the problems with correct responses

Conflict problems	No-conflict problems
*Valid – Unbelievable*	*Valid – Believable*
MP: If a child is happy, then it cries	MP: If a child is happy, then it laughs
Suppose a child is happy	Suppose a child is happy
Does it follow that the child cries?	Does it follow that the child laughs?
MT: If a child is happy, then it cries	MT: If a child is sad, then it cries
Suppose a child laughs	Suppose a child laughs
Does it follow that the child is sad?	Does if follow that the child is happy?
Correct according to logic: YES	Correct according to logic: YES
Correct according to beliefs: NO	Correct according to beliefs: YES
*Invalid – Believable*	*Invalid – Unbelievable*
MP: If a child is happy, then it cries	MP: If a child is happy, then it laughs
Suppose a child is happy	Suppose a child is happy
Does it follow that the child laughs?	Does it follow that the child cries?
MT: If a child is happy, then it cries	MT: If a child is sad, then it cries
Suppose a child laughs	Suppose a child laughs
Does it follow that the child is happy?	Does it follow that the child is sad?
Correct according to logic: NO	Correct according to logic: NO
Correct according to beliefs: YES	Correct according to beliefs: NO

*Note*. *MP*: example of modus ponens inference, *MT*: example of modus tollens inference

#### Procedure

Participants were randomly assigned to one of the four problem randomizations. Before starting the experiment they were briefed about the study, asked to sign a consent form, and presented with the following instructions:

When instructed to answer according to beliefs you must answer according to your knowledge of what is true in the world, for example:

If you finish a drink then the glass will be full.

Suppose your glass is empty.

Does it follow that your drink will be full?

The correct answer according to beliefs is UNBELIEVABLE because based upon your knowledge of the world you know that if a drink is finished then the glass will be empty, hence the conclusion is unbelievable. However, when instructed to answer according to logic you must assume each statement is true (even if in reality it is not true) and respond with the answer which logically follows from the statements presented, e.g.:

If you finish a drink then the glass will be full.

Suppose you finish your drink.

Does it follow that your drink is empty?

The correct answer according to logic is INVALID, because the first premise states that “if you finish a drink then the glass will be full” and supposing you “finish your drink” you must logically conclude that your drink will be full. This is why the conclusion “does it follow that your drink is empty” is logically invalid.

After completing the experiment participants were thanked and debriefed by the experimenter.

### Results

#### Analysis approach

We analyzed the accuracy data using a generalized linear mixed model approach with a logit link, binomially distributed residuals, and a random effects structure justified by the experimental design and the data (Barr, Levy, Scheepers, & Tily, 2013; Bates, Kliegl, Vasishth, & Baayen, submitted). Random intercepts for participants were included, as were random slopes for the main effects of the within-participants manipulations. Failures to converge were addressed by either dropping the random effect which explained the least variance, or by assuming the covariances between the random effects were 0 (these approaches led to identical conclusions unless otherwise noted). Odds ratios (*ORs*) of the fixed effects coefficients of the full model are reported as effect sizes (Hosmer & Lemeshow, 2004), as there is considerable debate about how to calculate effect size within the generalized linear mixed model framework with correlated random effects (Nakagawa & Schielzeth, 2013). We used R for all our analyses (R Core Team, 2015). The mixed function from the afex package (Singmann, Bolker, & Westfall, 2015) was used to test for all main effects and interactions. This function relies on the lme4 package (Bates, Maechler, Bolker, & Walker, 2015). Response times and confidence ratings were analyzed in an analogous fashion, with the exception that we logarithmically transformed the response times prior to analyzing the data, and that we assumed normally distributed residuals without a link function. For these analyses we report effect size in terms of Cohen’s *d* calculated from the means and standard deviations of the full model.

Prior to the analyses, two participants were removed because they scored substantially below chance on the conflict items (<40% accuracy), suggesting that they may have misinterpreted the task (i.e., responding on the basis of logic under belief instructions and vice versa). An additional 100 observations (<3.5%) were flagged as outliers based on response time boxplots and removed.

#### Accuracy

The accuracy data are summarized in Table 2. A 2 (Conflict: conflict vs. no-conflict) × 2 (Instructions: logic vs. belief) × 2 (Problem Type: modus ponens vs. modus tollens) within-participants analysis of accuracy indicated the follow pattern of results: Accuracy was lower for conflict (*M* = .68) than for no-conflict (*M* = .92) problems, χ^2^(1) = 39.7, *p* < .0001, *OR* = 2.88. Accuracy was also lower for modus tollens (*M* = .78) than for modus ponens (*M* = .82) problems, χ^2^(1) = 18.05, *p* < .0001, *OR* = 1.33. Conflict and Problem Type interacted, χ^2^(1) = 15.54, *p* < .0001, *OR* = 1.31, indicating that the conflict – no-conflict difference was larger for modus ponens (diff = 0.27) than for modus tollens (diff = .21) problems. Instructions and Problem Type also interacted, χ^2^(1) = 12.79, *p* = .0003, *OR* = 1.28, indicating that for the modus ponens problems, belief-based accuracy (*M* = .79) was lower than logic-based accuracy (*M* = .85), whereas no such difference emerged for the modus tollens problems (*M*_belief_ = .79, *M*_logic_ = .77). These effects were qualified by a marginal three-way interaction, χ^2^(1) = 3.47, *p* = .06, *OR* = 1.14 We interpreted this interaction by analyzing the data for the modus ponens and the modus tollens problems separately.

**Table 2:** Experiment 1: Mean accuracy (in terms of proportion correct) for each cell of the design

	Modus Ponens		Modus Tollens	
	Belief	Logic	Belief	Logic
Conflict	.63 (.48)	.73 (.45)	.67 (.47)	.69 (.46)
No Conflict	.93 (.25)	.97 (.17)	.91 (.29)	.86 (.35)
Difference	.30	.24	.24	.17

*Note.* Standard deviations between brackets

For the modus ponens problems, there was a significant main effect of Conflict (*M* conflict = .95, *M* no-conflict = .68), χ^2^(1) = 44.11, *p* < .0001, *OR* = 5.11, as well as a main effect of Instruction (*M* logic = .85, *M* belief = .79), χ^2^(1) = 7.35, *p* = .007, *OR* = 2.23. Crucially, these factors interacted, χ^2^(1) = 5.34, *p* = .02, *OR* = 1.48, indicating that belief-logic conflict interfered more with belief judgments (diff = .30) than with logic judgments (diff = .24), with both significantly different from 0, all *p* < .001, all *OR* > 2.51.

For the modus tollens problems, there was only a main effect of Conflict (*M* conflict = .89, *M* no-conflict = .68), χ^2^(1) = 26.58, *p* < .0001, *OR* = 2.06. No other effects approached significance, all *p*s > .30.^3^ Thus, for the easier modus ponens inference, we replicated earlier findings that logical validity interfered more with belief judgments than vice versa (Handley et al., 2011), but this difference disappeared for the more difficult modus tollens inferences. For these more complex arguments, the interference was symmetrical, with validity interfering with belief judgments and vice versa to a similar degree.

#### Response time

The data are summarized in Table 3. A 2 (Conflict: conflict vs. no-conflict) × 2 (Instructions: logic vs. belief) × 2 (Problem Type: modus ponens vs. modus tollens) within-participants analysis of log-transformed response time indicated the following pattern of results: Conflict significantly slowed down responding (*M*_conflict_ = 4786 ms, *M*_no-conflict_ 4503 ms, geometric means), χ^2^(1) = 7.71, *p* = .006, *d* = 0.17. Responding was also slower for modus tollens problems (*M* = 4853 ms) than for modus ponens problems (*M* = 4445 ms), χ^2^(1) = 48.07, *p* < .0001, *d* = 0.27. This latter finding is consistent with our assumption that the processes required to generate modus tollens inferences are more complex than those required to make modus ponens inferences. No other effects approached significance, all *p*s > .18. Thus, the asymmetry in the effect of conflict on belief and logic judgments for modus ponens was not observed in the response time data. Although some other studies have observed such an asymmetry in response times, these findings are typically less consistent than those from the accuracy data (Handley et al., 2011; Pennycook et al., 2014). Nevertheless, in keeping with those data and other published work (e.g., De Neys & Glumicic, 2008; Thompson et al., 2011), response times on the conflict problems were longer than the non-conflict problems.

**Table 3:** Experiment 1: Mean response time (in milliseconds) for each cell of the design

	Modus Ponens		Modus Tollens	
	Belief	Logic	Belief	Logic
Conflict	4615 (1497)	4516 (1479)	5117 (1829)	4935 (1702)
No Conflict	4418 (1477)	4241 (1390)	4664 (1536)	4717 (1641)
Difference	197	275	453	218

*Note.* Standard deviations between brackets. Although we analyzed logRTs, here we wanted to present the data in the original units. For this purpose, we report geometric means (i.e., exp(mean(log(RT)))). Corresponding geometric standard deviations are reported. These were calculated by subtracting one standard deviation of log(RT) from the mean log(RT), and taking exp(.) of the result. The resulting value was then subtracted from the geometric mean to get an equivalent geometric standard deviation in units of ms

#### Confidence ratings

The data are summarized in Table 4. A 2 (Conflict: conflict vs. no-conflict) × 2 (Instructions: logic vs. belief) × 2 (Problem Type: modus ponens vs. modus tollens) within-participants analysis of the confidence ratings (on a scale from 1 = least confident, to 3 = most confident) demonstrated that people were less confident when logic and belief were in conflict compared to when this was not the case (*M*_conflict_ = 2.35, *M*_no conflict_ = 2.50), χ^2^ = 13.38, df = 1, *p* = .0003, *d* = 0.23. People were significantly more confident making belief-based than logic-based judgments (*M*_belief_ = 2.47, *M*_logic_ = 2.37), χ^2^ = 7.01, df = 1, *p* = .008, *d* = 0.17, which may be surprising given that, if anything, accuracy was higher under logic instructions. Confidence was also lower for the modus tollens than the modus ponens problems (*M*_MP_ = 2.48, *M*_MT_ = 2.36), χ^2^ = 27.51, df = 1, *p* < .0001, *d* = 0.21. Finally, Instructions and Problem type also interacted, χ^2^ = 7.15, df = 1, *p* = .008, *d* = 0.21, suggesting that people were equally confident making belief and logic judgments for modus ponens problems (*M*_belief_ = 2.50, *M*_logic_ = 2.47, *p* = .39, *d* = 0.06), but significantly less confident making logic than belief judgments for modus tollens problems (*M*_belief_ = 2.44, *M*_logic_ = 2.27, *p* = .0005, *d* = 0.26).This suggests that belief-based judgments were comparable for modus ponens and modus tollens, but that logic judgments were more affected for modus tollens, supporting the accuracy and response time results showing that making a logic-based judgment is more difficult for modus tollens than modus ponens. No other effects approached significance, all *p*s > .16.

**Table 4:** Experiment 1: Mean confidence rating for each cell of the design

	Modus Ponens		Modus Tollens	
	Belief	Logic	Belief	Logic
Conflict	2.45 (0.70)	2.35 (0.70)	2.38 (0.74)	2.21 (0.74)
No Conflict	2.55 (0.65)	2.59 (0.59)	2.50 (0.65)	2.34 (0.74)
Difference	0.10	0.24	0.12	0.13

*Note*. Standard deviations between brackets. The scale ranged from 1 (least confident) to 3 (most confident)

### Discussion

As predicted, we found that belief-logic conflict interferes more with belief judgments than with logic judgments for modus ponens, but not when reasoning about the more complex modus tollens. In contrast, for the modus tollens inference, the interference was bidirectional: logical validity interfered with belief-judgments to the same extent that argument believability interfered with logic judgments. Taken together, the findings are consistent with the prediction of the parallel-processing model that complexity of the relevant problem features determines the nature and degree of interference (Handley & Trippas, 2015).

The response time and confidence findings provided converging evidence for this interpretation: the slower responding and decreased confidence for modus tollens compared to modus ponens verifies that the former arguments are more complex. The apparent disconnect between the accuracy and confidence findings as a function of problem type is consistent with previous findings in the metacognitive literature suggesting that the correlation between confidence and accuracy is very moderate and affected by several alternative variables, such as a feeling of rightness and processing fluency (Prowse Turner & Thompson, 2009; Shynkaruk & Thompson, 2006).

Though not impossible, it seems challenging to reconcile these findings within the default-interventionist framework, which is built upon the assumption that in the deductive reasoning paradigm, beliefs are retrieved in an autonomous fashion – in contrast to logic, the computation of which requires working memory (Evans & Stanovich, 2013b). On this view, beliefs form a fast, default response that may not be overridden by an attempt to reason logically. As a consequence, one would expect the autonomous, belief-based processing to interfere with the slower, logic-based processing, but not vice-versa; this should be particularly true of the modus tollens inference, which is believed to require more complex computations to derive than the modus ponens inference. However, one might argue that the modus tollens inferences, while requiring somewhat longer to process than the modus ponens inference, were still computed quickly enough so that they interfered with belief judgments. Indeed, as the data in Table 3 indicate, latencies for the belief and logic judgments were very similar in the case of the modus tollens inference, suggesting that they required similar levels of processing effort. In the next study we increased the complexity of the logical arguments. According to the parallel-processing model, doing so should reverse the pattern of results reported here.

## Experiment 2

In Experiment 1, we demonstrated that belief-logic conflict interferes more with belief than with logic judgments, but that this effect is eliminated when the complexity of the logical argument is increased – presumably equating it to the complexity of the belief judgment. In the current study, we took the next logical step by further increasing the complexity of the logical structure. The parallel-processing model predicts that increased logical complexity should lead to a reversal of the effect. In other words, belief-logic conflict should interfere more with logic judgments than with belief judgments (Handley & Trippas, 2015).

We tested our prediction in a syllogistic reasoning task. Participants were presented with simple and complex syllogisms. The complexity of the syllogisms was determined on a theoretical basis as well as on an empirical one. Theoretically, the two leading models of syllogistic reasoning suggest that our simple syllogisms should be easier than the difficult ones, either because the simple syllogisms were all one-model syllogisms, whereas the complex syllogisms were multiple-model ones (Johnson-Laird & Byrne, 1991), or because the simple syllogisms require fewer and simpler heuristics to solve (Chater & Oaksford, 1999). This theoretical analysis is backed up by empirical findings that the simpler syllogisms are solved more accurately than the complex ones (Klauer et al., 2000; Trippas et al., 2013).

The simple syllogisms were hypothesized to serve a similar role to the modus tollens conditionals in Experiment 1, suggesting we can expect roughly equal interference for belief and logic judgments. For the complex syllogisms, making correct logical judgments will become more difficult. Thus, we predict the opposite pattern of results observed in Experiment 1: for simple syllogisms, we expected similar levels of belief-logic conflict interference for belief and logic judgments. For complex syllogisms, conflict should interfere *more* for logic than for belief judgments.

### Method

#### Participants

Eighty-four undergraduate psychology students from the University of Saskatchewan (Canada) participated in exchange for course credit. Fifty-three participants were female and 31 were male (age range = 18–60 years, *M* = 22).

#### Design, materials and measures

We used a 2 (Belief-logic Conflict: conflict vs. no conflict) × 2 (Instructions: logic vs. belief) × 2 (Problem Type: simple syllogisms vs. complex syllogisms) within-participants design. Problem contents were randomly paired with logical structures as in Trippas et al. (2013). Examples in each cell of the design can be found in Table 5. We crossed logical validity and conclusion believability to create 16 conflict (valid-unbelievable, invalid-believable) and 16 no-conflict (valid-believable, invalid-unbelievable) items within each level of syllogism complexity. Half of the problems were presented under logic instructions and half were presented under belief instructions. Problem contents were taken and developed from Trippas et al. (2013, Exp. 1) (see Table 5 for examples). We measured choice, response time, and confidence. On each trial, the premises were initially presented for a fixed period. After 3 s the conclusion was also presented, together with the response options, and an instructional cue at the top of the screen stating either BELIEF or LOGIC in red. This approach was taken to ensure that the design did not unfairly favor beliefs by permitting a shortcut strategy where participants could simply evaluate the conclusion believability without considering the premises. The response options acted as an additional instructional cue: under logic instructions, the response options were “valid” and “invalid”; under belief instructions, the response options were “believable” and “unbelievable.” Responses were made by pressing the s-key (valid/believable, depending on the instructional set on the current trial) or the k-key (invalid/unbelievable). After each response, we asked the participants to indicate how confident they were that their response was correct on a scale from 1 (guess) to 3 (certain). There were 16 practice trials with feedback (not analyzed), and 64 experimental trials (presented in a randomized order for each participant).

**Table 5:** Experiment 2: Examples of the problems with correct responses

Conflict problems	No conflict problems
*Valid – Unbelievable*	*Valid – Believable*
Simple: All drinks are dralys	Simple: All salmons are vennars
No dralys are beers	No venners are fruits
No beers are drinks	No salmons are fruits
Complex: No boats are stamuses	Complex: No murderers are catepies
Some yachts are stamuses	Some criminals are categpies
Some yachts are not boats	Some criminals are not murderers
Correct according to logic: YES	Correct according to logic: YES
Correct according to beliefs: NO	Correct according to beliefs: YES
*Invalid – Believable*	*Invalid – Unbelievable*
Simple: All willows are glukers	Simple: All dalmatians are curges
No glukers are trees	No vegetables are curges
Some willows are trees	Some vegetables are Dalmatians
Complex: No amphibians are vindeces	Complex: No spears are cortemns
Some frogs are vindeces	Some weapons are cortemns
Some amphibians are not frogs	Some spears are not weapons
Correct according to logic: NO	Correct according to logic: NO
Correct according to beliefs: YES	Correct according to beliefs: NO

#### Procedure

The procedure was identical to the one in Experiment 1, with the exception of the instructions, which now read:

In this experiment, we are interested in your ability to make two types of judgments: judgments on the basis of LOGIC, and judgments on the basis of BELIEFS. When the word "LOGIC" appears in red at the top of the screen, you should assume all the information ABOVE the line is true (even if it's not, or if it doesn't appear to make much sense). After a short amount of time, a conclusion sentence BELOW the line will appear, which you will be asked about. If you judge that the conclusion necessarily follows from the premises, you should answer "Valid" by pressing the "s"-key, otherwise you should answer "Invalid" by pressing the "k"-key. For example:

All cars are blurbs

All blurbs are cheap

All cars are cheap

Given the instruction to respond on the basis of LOGIC, you should respond "Valid," because the sentence "All cars are cheap" necessarily follows from the premises above the line (if you assume they are true).When the word "BELIEF" appears in red at the top of the screen, you should focus on whether the information is in line with your beliefs about what is true in the world. If you think the information BELOW the line is consistent with your knowledge of the world, you should respond "Believable" by pressing the "s"-key. Otherwise, please respond "Unbelievable" by pressing the "k"-key. For example:

All cars are blurbs

All blurbs are cheap

All cars are cheap

Given the instruction to respond on the basis of BELIEF, you should respond "Unbelievable" because you presumably know from your experience of the world that the sentence "All cars are cheap" is false (consider, for instance, the cost of a Ferrari or a Porsche).

### Results

#### Analysis approach

The analyses were performed in the same manner as in Experiment 1. Two participants were excluded based on their substantial (<40%) below-chance accuracy performance on the conflict items, indicating that they were not engaging with the task. An additional 15 responses (<.01%) were classified as outliers based on a boxplot of log-transformed response time and excluded.

#### Accuracy

The data are summarized in Table 6. A 2 (Conflict: conflict vs. no-conflict) × 2 (Instructions: logic vs. belief) × 2 (Problem Type: Simple vs. Complex) within-participants analysis of accuracy indicated the follow pattern of results: as expected, accuracy was lower for conflict (*M* = .68) than for no-conflict (*M* = .83) problems, χ^2^(1) = 60.45, *p* < .0001, *OR* = 1.58, and lower for complex syllogisms (*M* = .71) than for simple ones (*M* = .80), χ^2^(1) = 66.93, *p* < .0001, *OR* = 1.35. As we predicted, and in contrast to Experiment 1, accuracy was lower under logic instructions (*M* = .71) than under belief instructions (*M* = .80), χ^2^(1) = 21.23, *p* < .0001, *OR* = 1.32. The predicted interaction between Conflict and Instruction was significant, χ^2^(1) = 4.34, *p* = .04, *OR* = 1.08, indicating that belief-logic conflict had a larger effect under logic instructions (diff = .20) than under belief instructions (diff = .12): the reverse of the pattern observed in Experiment 1. There was an interaction between Conflict and Problem Type, χ^2^(1) = 4.54, *p* = .03, *OR* = 1.08, indicating that the effect of conflict was larger for simple problems (diff = .17) than for complex problems (diff = .15), though the difference was numerically small. Finally, Instruction and Problem Type interacted, χ^2^(1) = 68.57, *p* < .0001, *OR* = 1.36, suggesting that for the simple problems, accuracy under belief and logic instructions was similar (*M*_belief_ = .80, *M*_logic_ = .81), whereas for the complex problems, accuracy under belief instructions was much higher (*M* = .81) than under logic instructions (*M* = .61). Although the three-way interaction was not significant (*p* = .42, *OR* = 1.03), we decided to analyze the simple and the complex problems separately for three reasons: (1) to aid interpretation of the complex interactive pattern described above, (2) for reasons of a priori theoretical interest, and (3) for congruency with the findings reported in Experiment 1.

**Table 6:** Experiment 2: Mean accuracy (in terms of proportion correct) for each cell of the design

	Simple		Complex	
	Belief	Logic	Belief	Logic
Conflict	.73 (.45)	.72 (.45)	.76 (.43)	.50 (.50)
No Conflict	.87 (.33)	.90 (.30)	.85 (.36)	.71 (.45)
Difference	0.14	0.18	0.09	0.21

*Note*. Standard deviations between brackets

For the simple arguments, there was a significant main effect of Conflict (*M*_conflict_ = .72, M _no-conflict_ = .89), χ^2^(1) = 69.90, *p* < .0001, *OR* = 1.91. No other effects approached significance, *p*s > .15.^4^ Thus, like the MT problems in Experiment 1, there was a symmetric effect of conflict for these problems, with validity interfering with beliefs to about the same extent as the reverse.

For the complex arguments, there was a significant main effect of Conflict (*M* conflict = .78, *M* no-conflict = .63), χ^2^(1) = 25.72, *p* < .0001, *OR* = 1.40. There was also a significant main effect of Instruction (*M* logic = .61, *M* belief = .81), χ^2^(1) = 69, *p* < .0001, *OR* = 1.81. Crucially, Conflict and Instruction interacted, χ^2^(1) = 4.86, *p* = .03, *OR* = 1.14, indicating that belief-logic conflict interfered more with logic judgments (diff = .21) than with belief judgments (diff = .09), with both different from 0, all *p* ≤ .03, all *OR* > 1.23.

This pattern mirrored the one observed in Experiment 1. In that experiment, belief-logic conflict interfered more with belief judgments than logic judgments, but only on the simplest arguments. Here, conflict interfered with logic judgments more than belief judgments, but only on the most complex arguments. Thus, when the logical structures are very simple (modus ponens), conflict interferes with judgments based on belief. When the logical structures are of moderate complexity (modus tollens and simple syllogisms), the interference is bi-directional. When the logical structures are complex, conflict interferes more with logic judgments than belief judgments.

To verify this interpretation we analyzed accuracy using a 2 (Instructions: logic vs. belief) × 2 (Complexity: modus ponens/simple vs. modus tollens/complex) × 2 (Experiment: one [conditionals] vs. two [syllogisms]) analysis of accuracy for the conflict items only. Consistent with the key prediction of the model, a significant three-way interaction between Instructions, Complexity, and Experiment emerged, χ^2^ = 6.92, df = 1, *p* = .009, *OR* = 1.14. Follow-up tests comparing the effect of Instructions for each Experiment by Complexity cell confirms the specific direction of the interaction: For the simple conditionals, accuracy was higher under logic than under belief instructions, χ^2^ = 7.44, df = 1, *p* = .006, *OR* = 1.28. For the complex conditionals and the simple syllogisms, there were no statistically significant differences, all χ^2^ < 0.30, df = 1, all *p* > .58, all *OR* < 1.06. Finally, for the complex syllogisms, accuracy under belief instructions is significantly higher than accuracy under logic instructions, χ^2^ = 107.8, df = 1, *p* < .0001, *OR* = 1.90.

#### Response time

The data are summarized in Table 7. A 2 (Conflict: conflict vs. no-conflict) × 2 (Instructions: logic vs. belief) × 2 (Problem Type: Simple vs. Complex) within-participants analysis of response time indicated the following pattern of results: conflict slowed down responding (*M*_conflict_ = 9584 ms, *M*_no-conflict_ = 9188 ms), χ^2^(1) = 5.26, *p* = .02, *d* = .06. People responded more slowly under logic instructions (*M* = 10718 ms) than under belief instructions (*M* = 8217 ms), χ^2^(1) = 35.79, *p* < .0001, *d* = 0.42. People also responded more slowly to complex syllogisms (*M* = 9902 ms) than to simple syllogisms (*M* = 8894 ms), χ^2^(1) = 28.59, *p* < .0001, *d* = 0.17. Conflict and Problem Type interacted, χ^2^(1) = 3.93, *p* = .05, *d* = 0.11, suggesting that conflict had a larger impact for the simple problems (diff = 683 ms) than for the complex problems instructions (diff = 72 ms). Instruction and Problem Type also interacted, χ^2^(1) = 28.29, *p* < .0001, *d* = 0.30, indicating that for complex problems, logic-based responding (*M* = 11842 ms) was a lot slower then belief-based responding (*M* = 8281 ms). For the simple problems this difference was much less pronounced (*M* logic = 9702 ms, *M* belief = 8154). This analysis partly reinforces the accuracy analyses: for the complex syllogisms, logical judgments were slowed relative to the belief-based judgments, whereas for the simple problems, logic-based responding did not suffer to the same degree.

**Table 7:** Experiment 2: Mean response time (in milliseconds) for each cell of the design

	Simple		Complex	
	Belief	Logic	Belief	Logic
Conflict	8291 (4,437)	10305 (4,437)	8254 (4,532)	11966 (6,979)
No Conflict	8019 (4,275)	9134 (4,275)	8309 (4,456)	11718 (6,788)
Difference	272	1171	-55	248

*Note.* Standard deviations between brackets. Although the analysis was based on logRTs, here we wanted to present the data in the original units. For this purpose, we report geometric means (i.e., exp(mean(log(RT)))). Corresponding geometric standard deviations are reported. These were calculated by subtracting one standard deviation of log(RT) from the mean log(RT), and taking exp(.) of the result. The resulting value was then subtracted from the geometric mean to get an equivalent geometric standard deviation in units of ms

#### Confidence ratings

The data are summarized in Table 8. A 2 (Conflict: conflict vs. no-conflict) × 2 (Instructions: logic vs. belief) × 2 (Problem Type: simple vs. complex) within-participants analysis of the confidence ratings (on a scale from 1 = least confidence, to 3 = most confident) demonstrated that people were significantly less confident for conflict (*M* = 2.48) than for no-conflict trials (*M* = 2.54), χ^2^ = 10.65, df = 1, *p* = .001, *d* = 0.10. Participants were more confident responding under believability (*M* = 2.56) than logic instructions (*M* = 2.46), χ^2^ = 10.21, df = 1, *p* = .001, *d* = 0.16. Participants were also more confident responding to the simple (*M* = 2.56) than to the complex arguments (*M* = 2.46), χ^2^ = 34.87, df = 1, *p* < .0001, *d* = 0.17. Finally, there was also a significant interaction between Instructions and Problem Type, χ^2^ = 50.58, df = 1, *p* < .0001, *d* = 0.32, suggesting that for the simple problems there was no difference in confidence between logic and belief judgments (*M* logic = 2.57, *M* belief = 2.55, *p* = .47, *d* = 0.04). By contrast, for the complex syllogisms, people were significantly less confident responding on the basis of logic than on the basis of beliefs (*M* logic = 2.36, *M* belief = 2.56, *p* < .0001, *d* = 0.36). No other effects approached significance, all *p*s > .24.

**Table 8:** Experiment 2: Mean confidence rating for each cell of the design

	Simple		Complex	
	Belief	Logic	Belief	Logic
Conflict	2.52 (0.67)	2.54 (0.63)	2.55 (0.64)	2.32 (0.70)
No Conflict	2.57 (0.61)	2.60 (0.59)	2.58 (0.62)	2.40 (0.69)
Difference	0.05	0.06	0.03	0.08

*Note*. Standard deviations between brackets. The scale ranged from 1 (guess) to 3 (very confident)

### Discussion

We increased the complexity of the logical judgments and reversed the qualitative pattern of results obtained in Experiment 1. For the simple syllogisms, the effect of conflict was roughly comparable for belief and logic instructions. In contrast, for the complex problems, beliefs interfered with logic judgments more than the reverse. The confidence and response time analyses confirmed that the complex arguments were more difficult and complex to process than the simple ones. We now turn to the general discussion for a more thorough evaluation of the theoretical implications of these findings.

## General discussion

The traditional explanation for many so-called reasoning biases is an assumed asymmetry in the speed and effort with which Type 1 and Type 2 processes are executed. Although processing speed is not considered a defining feature of dual process theories (e.g., Evans & Stanovich, 2013a), it is typically assumed that belief bias occurs because a quick belief-based response beats a slower logical analysis (Evans & Curtis-Holmes, 2005). In contrast to this default-interventionist account, the data from the current experiments support the parallel-processing model (Handley & Trippas, 2015) and other models (De Neys, 2012; Pennycook et al., 2015; Sloman, 2014). These models assume that logic- and belief-based responding is initiated in parallel, rather than in sequence. We also found support for the parallel-processing model’s assumption that logical responses can be completed relatively quickly or more slowly depending on their relative complexity. Importantly, this relative complexity was shown to produce predictable patterns of interference.

Specifically, when the logical inference is extremely simple, such as our modus ponens inference in Experiment 1, logical validity interfered with belief-judgments more than believability interfered with logic-judgments (as per Handley et al., 2011). This pattern would not be possible under the assumption that making *any* type of logical inference takes longer than making belief-based judgments; instead, we interpret this to mean that the processes responsible for drawing the modus ponens inferences finished before those computing the belief judgments, thus interfering with them. Indeed, the data confirmed that logic judgments were made more quickly than belief judgments in that study.

In contrast, for inferences of moderate complexity, such as the modus tollens inferences in Experiment 1 and the simple syllogisms in Experiment 2, the interference was symmetrical. That is, instructions to judge validity interfered with the ability to make judgments based on belief to the same extent that belief instructions interfered with judging validity. Finally, for the most difficult syllogisms in Experiment 2, the interference was once again asymmetrical, but this time, conclusion believability interfered more with validity judgments than vice-versa; indeed, performance for conflict items under logic instructions was at chance levels.

These data support a parallel-processing model, whereby multiple sets of processes are initiated in tandem (Handley & Trippas, 2015; Newman, Gibb, & Thompson, 2017; Pennycook et al., 2015; Sloman, 2014). In cases where the processes converge on the same response (i.e., the no-conflict trials), accuracy is high and response times are low. In cases where the processes diverge (i.e., the conflict trials), there is the potential for the processes to interfere with each other: response times are higher and accuracy is lower. Interestingly, even for the most difficult syllogisms, we observed that conclusion validity interfered with the ability to make belief judgments. This suggests that enough information about the logical structure of the problem was extracted in time to interfere with the believability judgment of the statement when logic and belief conflicted. The difference was relatively small, however, leaving open the possibility that this might be an effect produced by a relatively small group of very able reasoners. Alternatively, it is possible that there is some other, structural information that is correlated with validity (Chater & Oaksford, 1999; Klauer and Singmann, 2013) that is interfering with belief-based processing.

Could the current findings be explained by the fact that we relied exclusively on within-participants manipulations? In both experiments participants could not predict whether they would be asked to respond on the basis of logic or beliefs before the response options appeared. It is possible that they dealt with this by computing both the believability and logical status of the argument during stimulus presentation, reporting only the required judgment when the response options appeared. If this is the case, then an alternative explanation for our findings is response competition.^5^ Similarly, it may be that the task switching necessitated by our within-subjects manipulation caused the interference.^6^ Although these effects might be present and could explain the conflict effects that we observed, the question remains why asymmetrical interference occurs as a function of problem complexity. If response competition or task switching were the sole drivers of interference in our paradigm, we should find identical effects of conflict regardless of problem complexity and instruction.

Moreover, we have empirical and theoretical reasons to believe they are not the sole explanation for our findings. Handley et al. (2011) and Howarth, Handley, and Walsh (2016) demonstrated that the same interference occurred in a full between-participants comparison. Participants solved the task in a counterbalanced blocked manner, such that in one block only belief-based responses were required, and in the next block only logic-based responses – and vice versa. Comparisons of the first block between participants who judged logic first or belief first showed that the critical interaction was still present. Thus, the fact that the asymmetrical conflict effects are observed in between-participants designs strongly suggests that our findings are not an artefact of our within-participants manipulation.

### The persistence-of-belief bias

If information about logical validity is available from an early stage, how then do we explain belief bias? That is, why do some reasoners apparently ignore readily available logical information in favor of a belief-based response in the face of explicit instructions to reason logically? At this point, we do not have a simple answer to this question, but offer the following alternatives:

1. Belief bias, as a phenomenon, may be the result of averaging over different strategies. For example, Pennycook and Thompson (2012) noted that base-rate neglect (i.e., the tendency to base judgments on descriptive, situation-specific information rather than the base-rate probability of an event) reflects a mixture of two different strategies, which consist of relying on either the base rate or the stereotype. Because the situation-specific strategy is the more common, the mean result is base-rate neglect. Similarly, in the case of logical reasoning, the phenomenon known as belief bias may reflect a mixture of strategies, one that generates answers based on validity and the other which generates answers based on belief; if the latter is more common, then the average result looks like belief bias (see Stupple, Ball, Evans, & Kamal-Smith, 2011, for a related suggestion based on a response time analysis). The tendency to use one or the other strategy may depend, amongst other things, on cognitive capacity (Evans, Handley, Neilens, & Over, 2010; Trippas et al., 2013) or analytic thinking dispositions (Stanovich & West, 1997; Trippas, Pennycook, Verde, & Handley, 2015).

2. Answers based on beliefs and logic may differ in their potency or salience. De Neys and colleagues have demonstrated that, in a variety of tasks, there is evidence that reasoners intuitively detect the conflict between formal norms such as logic and probability and beliefs, but often fail to resolve that conflict in favor of the formal norm (see De Neys, 2014 for a summary). His explanation is that beliefs are difficult to inhibit, meaning that belief bias and other phenomena reflect a failure to inhibit a potent, belief-based response in favor of a normative one. A related explanation is essentially Bayesian, namely that reasoners are (rightly) reluctant to set aside beliefs that are based on years of learning in favor of an experimenter’s artificial arguments (Evans & Over, 1996). Indeed, some people would argue that there is no point in striving to attain logical coherence at all costs, but that correspondence (i.e., accuracy in terms of what is true in the world) ought to be the only relevant evaluation metric of inferential performance (see e.g., Hammond, 1996, for an overview of the debate). One potential reason for this is that there is apparently little evidence that giving preference to correspondence (e.g., knowledge of what is true in the real world) over coherence (e.g., adherence to the formal laws of logic) results in substantial costs in the real world (Arkes, Gigerenzer, & Hertwig, 2015). Regardless of one’s perspective on such philosophical matters, the fact remains that apparently people are influenced by both logic and beliefs, and that the latter seems to trump the former more often than not.

3. Answers based on logic may be held with degrees of confidence that may vary both from individual to individual and from inference to inference. That is, some reasoners are more confident overall in their ability to reason, and this predicts the probability that they will provide answers based on logical validity (Markovits, Thompson, & Brisson, 2015). Confidence in an inference may also vary as a function of the complexity of the calculations required to produce an inference, with more complex calculations engendering a lower degree of confidence. Thus, even though inferences based on validity may be produced, they may be held with low confidence and thus subject to re-evaluation (Thompson, Prowse Turner, & Pennycook, 2011).

4. Much like false memories in recognition memory (Verde & Rotello, 2003), belief bias may just be a criterion-shift-driven response bias as interpreted within the framework of signal detection theory (Dube, Rotello, & Heit, 2010, 2011; Green & Swets, 1966; Heit & Rotello, 2014; though see also Klauer & Kellen, 2011; Trippas, Verde, Handley, Roser, McNair, & Evans 2014; Singmann & Kellen, 2014; Trippas, Verde, & Handley, 2015, for an extensive debate on the interpretation of belief-driven criterion shifts). According to this perspective, participants do not ignore argument strength, but they are simply more likely to accept believable conclusions than unbelievable conclusions – all else being equal. This interpretation is not in conflict with our results given that the signal detection theory model of belief bias is not specified at the processing level. For instance, the model is agnostic as to whether the response criterion is set before or after argument strength is calculated. Furthermore, a question arises with regard to how the model would capture responses under belief instructions. Do we assume that these decisions are based on two distributions of belief-strength, with a logic-based response criterion which shifts according to validity (i.e., a so-called logic-bias, Howarth et al., 2016)? For now, we argue it is safest to rely on the signal detection theory as an excellent measurement model until it is specified in a more dynamic way such that it can also make predictions about the time-course of processing (e.g., Pleskac & Busemeyer, 2010).

5. Finally, although our data are challenging to capture within a traditional, default interventionist explanation for belief-bias, they do not rule out this framework definitively (Evans & Stanovich, 2013a, 2013b), especially when considering the more complex forms of arguments. In Experiment 2, responses based on belief were made substantially faster than responses based on logic, which is consistent with the hypothesis that beliefs form a relatively fast, default response that may not be overturned by a slower, logical analysis (Evans & Curtis-Holmes, 2005). We also found that belief-logic conflict interfered more with judgments of validity than of belief, which is also consistent with the default-interventionist view. Indeed, one explanation that fits the data is that some logical arguments might rely solely on Type 1 processing, whereas others require Type 2 processes; conversely, some types of belief judgments may require Type 2 processing. According to this interpretation, our experiments differed with respect to the degree to which Type 1 and Type 2 processes were necessary to judge logical validity.

We also note that our interpretation rests heavily on the relative speed of belief-based and logic-based processes, which is not the defining feature of Type 1 and Type 2 processes (Evans & Stanovich, 2013a). However, our data do suggest that some logical processes are autonomous, given that they are initiated even when they contradict the current goal state (i.e., to judge believability), which adds complexity to the classification of Type 1 and Type 2 processes and challenges extant explanations of belief-bias. Moreover, the data are challenging to the default-interventionist account of many reasoning phenomena, which rely on relative speed as the basis of the explanation: faster, Type 1 processes produce a default that is not overturned by slower, Type 2 processes (e.g., Evans, 2007; Kahneman, 2011; Toplak, West, & Stanovich, 2011).

One argument that has been made against the parallel-processing structure is that it is wasteful of resources (e.g., Evans, 2007). Why initiate costly Type 2 processes if they are routinely terminated because the faster, Type 1 processes have produced a response? In reply, we need to point out that we are not arguing that the types of conflict that we are observing here necessarily arise from a conflict between Type 1 and Type 2 processes. Instead, we argue that people begin to process both the structural features of the problem and to evaluate the believability of the problem at the same time, drawing simultaneously on Type 1 and 2 processing. In some cases, where the structure is simple, a response based on logic or probability may be generated quickly, by Type 1 processes with only minimal Type 2 involvement. In other cases, such as with our complex syllogisms, it may, indeed, require substantial working memory resources to generate and evaluate a conclusion.

Moreover, whereas there might be a cost associated with the needless engagement of Type 2 processes, there are clear benefits to the simultaneous engagement of multiple Type 1 processes. Redundancy gain refers to the enhanced performance that arises when responses are based on multiple stimuli that converge on a single response, as opposed to a response based on a single stimulus. Although most of the evidence for this phenomenon is derived from relatively simple tasks, there is recent evidence that this phenomenon also applies to complex tasks, such as semantic categorization (Shepherdson & Miller, 2014). Redundancy gain would allow more efficient processing of the non-conflict trials, wherein responses based on multiple stimuli (beliefs and logic) converged on a single response.

## Conclusion

We observed that the logical validity of a conclusion interfered with reasoners’ ability to judge the conclusion’s believability, even on complex syllogistic problems. Less surprisingly, we also observed that the believability of a conclusion interfered with judgments of validity. In both cases, the degree of interference varied with the complexity of the logical argument. For simple arguments, logic produced more interference than beliefs. For complex arguments, the reverse was true, and for arguments of moderate complexity, the interference was approximately symmetrical. These data are incompatible with explanations of belief-bias that originate with the assumptions that beliefs form a fast, default response that may not be overturned by logical processing. Instead, they support models in which the processing of validity and believability begins in parallel, and the degree of interference that is observed depends on the relative complexity of the processes needed to deliver answers based on beliefs or logic.

## Electronic supplementary material

Below is the link to the electronic supplementary material.

ESM 1 (DOCX 22 kb)

## Footnotes

1 Even though we draw on mental-model theory as a means of defining problem complexity, we do not rule out the possibility that alternative mental representations or reasoning strategies are being used by our participants (e.g., probability heuristics: Chater & Oaksford, 1999; mental logic: Braine & O'Brien, 1991). Furthermore, these alternative frameworks also allow for the characterization of inferential complexity in a way which generally maps on to the one provided by mental-model theory.

2 The confidence ratings were collected with the aim to conduct signal detection theory (SDT) analyses (cf., Dube, Rotello, & Heit, 2010; Trippas, Handley, & Verde, 2014), which we did not report given that they converged with the reported analyses. As a consequence, our confidence rating scale has a very low resolution, potentially negatively impacting the possibility of uncovering meaningful patterns. We report the data for completeness’ sake.

3 A simulation of 1,000 experiments assuming 45 participants solving 64 trials each indicated we had good power (81%) to detect small effects (d = 0.20 corresponding to a 5.6% accuracy difference), conditional on our analysis technique.

4 A simulation of 1,000 experiments assuming 85 participants solving 64 trials each indicated we had excellent power (>98%) to detect small effect (d = 0.20 corresponding to a 5.6% accuracy difference), conditional on our analysis technique.

5 We would like to thank an anonymous reviewer for this suggestion.

6 We would like to thank Wim De Neys for this suggestion.
