# Supplementary material for: When fast logic meets slow belief: Evidence for a parallel-processing model of belief bias
Source: Mem Cognit. 2016 Dec 27;45(4):539–52. doi: 10.3758/s13421-016-0680-1 (PMC5432582; doi:10.3758/s13421-016-0680-1)
Supplement: Supplementary file 1 — (DOCX 22 kb) [file 13421_2016_680_MOESM1_ESM.docx]

**Supplementary Materials**

**Conflict Detection and Cognitive Decoupling**

Studying the well-known phenomenon of base-rate neglect (Kahneman & Tversky, 1973), Pennycook, Fugelsang, and Koehler (2015) demonstrated that *conflict detection* and *cognitive decoupling* are two experimentally dissociable type 2 processes. According to their dual process model of analytic engagement, there are three stages of processing during reasoning. During stage 1, type 1 processes cue a number of initial responses (IR_1_, IR_2_, …, IR_n_; ranked in decreasing salience) based on various problem cues, some structure-based (e.g., logic and probability), others knowledge-based (e.g., knowledge and beliefs about the world and stereotypes). During stage 2, a conflict between these various cued response potentials may or may not be detected. This is operationalized by subtracting correct no-conflict response time (RT) from incorrect conflict RT. During the final stage, if a conflict was present and detected, people may engage in additional processing to inhibit and override the initial response (i.e., cognitive decoupling) allowing them to give an alternative (possibly correct) response. This is operationalized by subtracting correct no-conflict RT from correct-conflict RT. Although the three-stage dual process model differs from the parallel processing model introduced in the current paper, it is nevertheless interesting to interpret a subset of the present findings through the lens of the three-stage model for concordance with the wider literature on dual processing and conflict detection. We first compare conflict detection as a function of argument complexity.

De Neys (2014) predicted that conflict detection should be diminished for complex compared to simple problems, given that it seems unlikely that laymen are intuitively sensitive to the logical validity of very complex syllogisms and conditionals. We manipulated complexity in our studies, allowing us to test this proposal by calculating and comparing the conflict detection index (ConfDet) under logic instructions for the MP and MT conditionals, as well as for the simple and complex syllogisms. Confirming De Neys’ prediction, it is indeed the case that conflict detection is more prevalent for simple than for complex syllogisms (ConfDet _simple_ = 2054 ms vs. ConfDet _complex_ = 525 ms), *t*(78) = 1.69, *p* = .048. In the context of conditional reasoning, the finding went in the predicted direction descriptively (ConfDet _MP_ = 452 ms vs. ConfDet _MT_ = 58 ms), if not statistically, *t*(37) = 1.07, *p* = .15 (both tests one-tailed and within-subjects). Next, we test whether the cognitive decoupling differs depending on whether logic or belief instructions are provided.

Based on the received view of dual process theory (e.g., Evans & Stanovich, 2013) one might predict that – all else being equal – cognitive decoupling is more effortful under logic instructions than under belief instructions. We compared the cognitive decoupling index (CogDec) for the logic and belief instructions, separately for the conditionals and syllogisms. Consistent with the traditional dual process prediction, for syllogisms the cognitive decoupling effect was larger under logic instructions than under belief instructions (CogDec logic = 1312 ms vs. CogDec belief = 435 ms), *t*(80) = 2.75, *p* = .007. However, for the conditional arguments the effect was reversed, with a larger cognitive decoupling effect under belief than under logic instructions (CogDec logic = 294 ms vs. CogDec belief = 864 ms), *t*(36) = 2.62, *p* = .013.
